# A General G1/S-Phase Cell-Cycle Control Module in the Flowering Plant *Arabidopsis thaliana*

**DOI:** 10.1371/journal.pgen.1002847

**Published:** 2012-08-02

**Authors:** Xin'Ai Zhao, Hirofumi Harashima, Nico Dissmeyer, Stefan Pusch, Annika K. Weimer, Jonathan Bramsiepe, Daniel Bouyer, Svenja Rademacher, Moritz K. Nowack, Bela Novak, Stefanie Sprunck, Arp Schnittger

**Affiliations:** 1 Department of Molecular Mechanisms of Phenotypic Plasticity, Institut de Biologie Mole´culaire des Plantes, Centre National de la Recherche Scientifique, Université de Strasbourg, Strasbourg, France; 2 Trinationales Institut für Pflanzenforschung, Strasbourg, France; 3 Unigruppe am Max-Planck-Institut für Pflanzenzü chtungsforschung, Lehrstuhl für Botanik III, Universität zu Köln, Köln, Germany; 4 Cell Biology and Plant Biochemistry, University of Regensburg, Regensburg, Germany; 5 Department of Plant Systems Biology, VIB, Gent, Belgium; 6 Department of Plant Biotechnology and Bioinformatics, Ghent University, Gent, Belgium; 7 Oxford Centre for Integrative Systems Biology, Department of Biochemistry, University of Oxford, Oxford, United Kingdom; University of Arizona, United States of America

## Abstract

The decision to replicate its DNA is of crucial importance for every cell and, in many organisms, is decisive for the progression through the entire cell cycle. A comparison of animals versus yeast has shown that, although most of the involved cell-cycle regulators are divergent in both clades, they fulfill a similar role and the overall network topology of G1/S regulation is highly conserved. Using germline development as a model system, we identified a regulatory cascade controlling entry into S phase in the flowering plant *Arabidopsis thaliana*, which, as a member of the *Plantae* supergroup, is phylogenetically only distantly related to *Opisthokonts* such as yeast and animals. This module comprises the *Arabidopsis* homologs of the animal transcription factor E2F, the plant homolog of the animal transcriptional repressor Retinoblastoma (Rb)-related 1 (RBR1), the plant-specific F-box protein F-BOX-LIKE 17 (FBL17), the plant specific cyclin-dependent kinase (CDK) inhibitors KRPs, as well as CDKA;1, the plant homolog of the yeast and animal Cdc2^+^/Cdk1 kinases. Our data show that the principle of a double negative wiring of Rb proteins is highly conserved, likely representing a universal mechanism in eukaryotic cell-cycle control. However, this negative feedback of Rb proteins is differently implemented in plants as it is brought about through a quadruple negative regulation centered around the F-box protein FBL17 that mediates the degradation of CDK inhibitors but is itself directly repressed by Rb. Biomathematical simulations and subsequent experimental confirmation of computational predictions revealed that this regulatory circuit can give rise to hysteresis highlighting the here identified dosage sensitivity of CDK inhibitors in this network.

## Author Summary

In order to grow, multicellular organisms need to multiply their cells. Cell proliferation is achieved through a complex order of events called the cell cycle, during which the nuclear DNA is duplicated and subsequently distributed to the newly forming daughter cells. The decision to replicate the nuclear DNA is in many organisms crucial to progress through the entire cell cycle. Alterations of the cell cycle, especially at the entry point, can cause severe developmental defects and are often causal for maladies, such as cancer. Substantial work in yeast and animals has revealed the regulatory steps controlling S-phase entry. In contrast, relatively little is known about the plant cell cycle despite plants being one of the largest classes of living organisms and despite the importance of plants for human life, for instance as the basis of human nutrition. Our work presents a molecular framework of core cell-cycle regulation for entry into the DNA replication phase in the model plant Arabidopsis. We report here the identification of a regulatory cascade that likely functions in many plant cells and organisms. With this, we also provide an important basis for comparative analyses of cell-cycle control between different eukaryotes, such as yeast and mammals.

## Introduction

Understanding the mechanisms of plant growth and differentiation is an important task, given the global biomass of land plants with approximately 600 billion tons of carbon [1]. Although cell proliferation is a main determinant of growth, relatively little is known about cell-cycle regulation in plants in comparison to yeast or metazoans.

The typical eukaryotic cell cycle, as found also in plants, is divided into four phases: the S (synthesis) phase in which the nuclear DNA is replicated; the M (mitosis) phase in which sister chromatids are separated and distributed to the newly forming daughter cells; and two gap (G1 and G2) phases that separate the M and S phases. The control of the G1-to-S-phase transition is a key step in cell-cycle regulation because cells typically become committed to divide after they have replicated their DNA [2]–[4]. In all eukaryotes, S-phase entry is tightly regulated by various mechanisms, incorporating intrinsic information, such as nutrient status and developmental program, with extrinsic, environmental conditions, such as temperature.

Intrinsic and extrinsic cues are integrated through a complex control of the central driving force of cell-cycle progression, i.e. the cyclin-dependent kinases (CDKs). Only when the sum of the different input systems is positive, CDKs become activated and entry into the next cell-cycle phase will be promoted once a certain threshold of activity is reached [5]. The four major input systems that regulate CDK activity are binding of positive cofactors (i.e. cyclins) and negative regulators (i.e. CDK inhibitors), and positive and negative phosphorylation events (i.e. at threonine and/or tyrosine residues of the T- and the P-loop, respectively). All four modules are themselves under elaborate control, for instance through regulation of the protein stability of CDK inhibitors [6].

Work in yeast and metazoans has shown that these regulatory modules are typically wired so that the activated CDK complex promotes its activators, while inhibiting its counter players. This circuitry leads only to two stable steady states, inactive or active, that generate a biological switch. Through the feedback wiring, the system becomes buffered against small changes in regulator concentrations, namely the activator concentration must be higher to switch from G1 phase to S phase than to remain in the S phase. This property of the feedback wiring, called hysteresis, greatly reinforces the switch-like behavior and is important in many biological processes; in the case of cell-cycle regulation, it is thought to be critical to promote the unidirectional progression of the cell cycle.

In general, cell-cycle regulation appears to be conserved among eukaryotes. Analysis of the *Arabidopsis thaliana* genome has revealed that the majority of the core cell-cycle regulators known from yeast and/or metazoans is also present in plants, such as homologs of the transcriptional regulator E2F and its counter player Retinoblastoma (Rb) [7]. In particular, the general theme of the CDK-cyclin-regulated cell-cycle progression seems to be conserved. Functional analyses have shown that CDKA;1, the only homolog of Cdk1/Cdc2^+^/CDC28p, is required throughout the *Arabidopsis* life cycle 8–12. Nevertheless, the mammalian and plant cell-cycle control differs pronouncedly. For instance, the CDKA;1 regulation by phosphorylation and dephosphorylation through WEE1 and CDC25, respectively, is not used in the cell-cycle control of *Arabidopsis* [11], [13], [14]. In addition, cell-cycle regulators are present in plants that are unknown in animal or yeast model systems or are only very distantly related to their metazoan or microbial counterparts. The plant CDK inhibitors, represented by two plant-specific groups, the INHIBITOR/INTERACTOR OF CDK or KIP-RELATED PROTEIN (ICK/KRP) and the SIAMESE RELATED are both only very little similar to the animal CDK inhibitor p27^Kip1^ [15]–[18].

A paradigm for the importance of precise cell-cycle control is the generation of gametes in flowering plants during the plant-specific gametophytic life phase that starts after meiosis with the formation of four monocellular haploid spores [19]. The male gametophyte has been analyzed in detail because it is more easily accessible than the female part, especially the cell proliferation and differentiation of the microspore into a mature pollen grain. The microspore undergoes strictly two rounds of mitotic cycles [20]. A first division of the microspore (pollen mitosis I [PMI]) that is asymmetric results in a smaller generative cell that is engulfed by a larger vegetative cell. The vegetative cell will exit the cell cycle and the plant retinoblastoma homolog RETINOBLASTOMA RELATED 1 (RBR1) is required to terminate this lineage, because pollen with multiple vegetative cells are formed in *rbr1* mutants [21], [22]. The generative cell represents the beginning of the short male germline and will undergo one final division (PMII), leading to two sperm cells, whereas RBR1 seemingly restricts its cell proliferation and/or the sperm cells, as supernumerary sperm cells can be found in *rbr1* pollen [21], [22].

The CDKA;1 activity is of key importance for PMII. The *cdka;1* mutant pollen develops a vegetative cell similar to the wild type, but only one generative/sperm-like cell, see below [9], [10]. A similar phenotype was observed also in mutants of the *F-BOX-LIKE 17* (*FBL17*) gene [23], [24]. FBL17 was found to act as an adaptor protein in an SKP-CULLIN-F-BOX (SCF) complex and to mediate the degradation of KRP6 and KRP7 [24]. Consistently, mutants of *KRP6* could partially restore the second mitotic division in *fbl17* mutants [23]. KRP6 seems to be regulated during plant reproduction by at least one other mechanism involving the RING E3 ligases, RING-H2 group F 1a (RHF1a) and RHF2a, that also target KRP6 for degradation [25]. In *rhf1a rhf2a* double mutants, embryo sac development was arrested at early stages and, likewise, pollen development was defective at PMI and PMII. These gametophytic cell-cycle defects could be phenocopied by *KRP6* overexpression [25], consistent with previous experiments in which pollen mitosis in *Brassica napus* (rapeseed) was blocked by ectopic expression of *ICK1*/*KRP1* from *Arabidopsis* [26]. However, a detailed molecular genetic framework of cell-cycle control is still missing in flowering plants. Especially, cell-cycle control during female gametophyte development is far from being understood.

Here, we identified a regulatory cascade that functioned during all divisions in female and male gametophyte development. Subsequently, the biomathematical modeling of this network revealed that this circuitry can generate hysteresis. In this network, the CDK inhibitors are of central importance. We postulate that this cascade builds a general G1/S-phase module that probably operates in all cells of *Arabidopsis* and other plant species as well.

## Results

### CDKA;1 is present during female and male gametogenesis and specifically marks gametic cells

**Figure 1:**
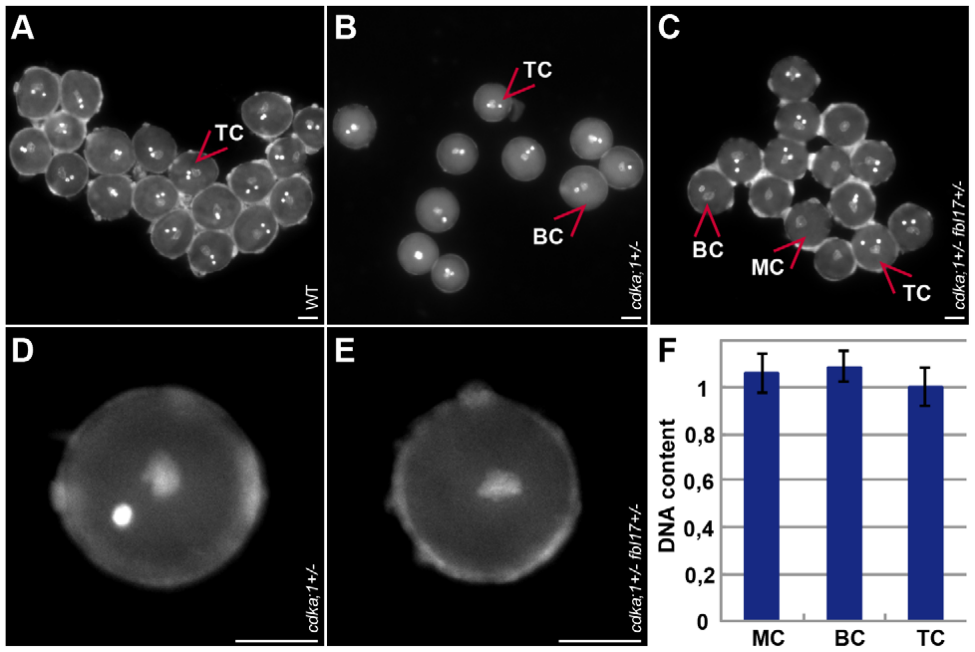
Pollen phenotypes of mutants for components of the G1/S phase control module. (A) Tricellular mature wild-type DAPI-stained pollen at anthesis (one vegetative cell enclosing two sperm cells). (B) DAPI-stained pollen at anthesis from heterozygous *cdka;1* mutant plants (similar to pollen from heterozygous *fbl17* mutants, data not shown) containing approximately 43% bicellular pollen (one vegetative cell and one sperm-cell-like cell) and 57% tricellular, wild-type-like pollen. (C) DAPI-stained pollen at anthesis from double heterozygous *cdka;1 fbl17* mutant plants carrying a hemizygous *Pro_CDKA;1_:CDKA;1:YFP* rescue construct (similar to pollen from *e2fa^−/−^ fbl17^+/−^* mutants, data not shown) and containing single-celled pollen grains (only one vegetative-like cell), in addition to bicellular (*cdka;1*/*fbl17*-like) and tricellular (wild-type-like) pollen. (D) Close-up of bicellular pollen as found in *cdka;1* or *fbl17* heterozygous plants. (E) Close-up of monocellular pollen grains as found in *cdka;1 fbl17* or *e2fa fbl17* double heterozygous mutants. (F) Quantification of DAPI-stained pollen. The DNA content of the single-celled pollen from *cdka;1^+/−^ fbl17^+/−^* or *e2fa^−/−^ fbl17^+/−^* double mutants reaches 1C, similarly to the vegetative nucleus in wild-type pollen and, thus, resides in a G1 phase.

As CDKA;1 is the only functional *Arabidopsis* homolog of the yeast Cdc2^+^/CDC28p protein, this kinase might plausibly be involved in cell-cycle control in every cell. This assumption was supported by hypomorphic *cdka;1* mutants, in which many, if not all, cells were affected, such as mitotically dividing cells in the epidermis of leaf primordia, as well as endoreplicating leaf hairs (trichomes) [8], [11], [27]. However, heterozygous null mutants were arrested or even delayed only in the second mitotic division during male gametogenesis, although the *CDKA;1* promoter was active throughout the male gametophyte development [9], [10], [28] (Figure 1A, 1B, 1D). Moreover, female gametogenesis that comprises three divisions was not affected at all in the heterozygous mutant.

**Figure 2:**
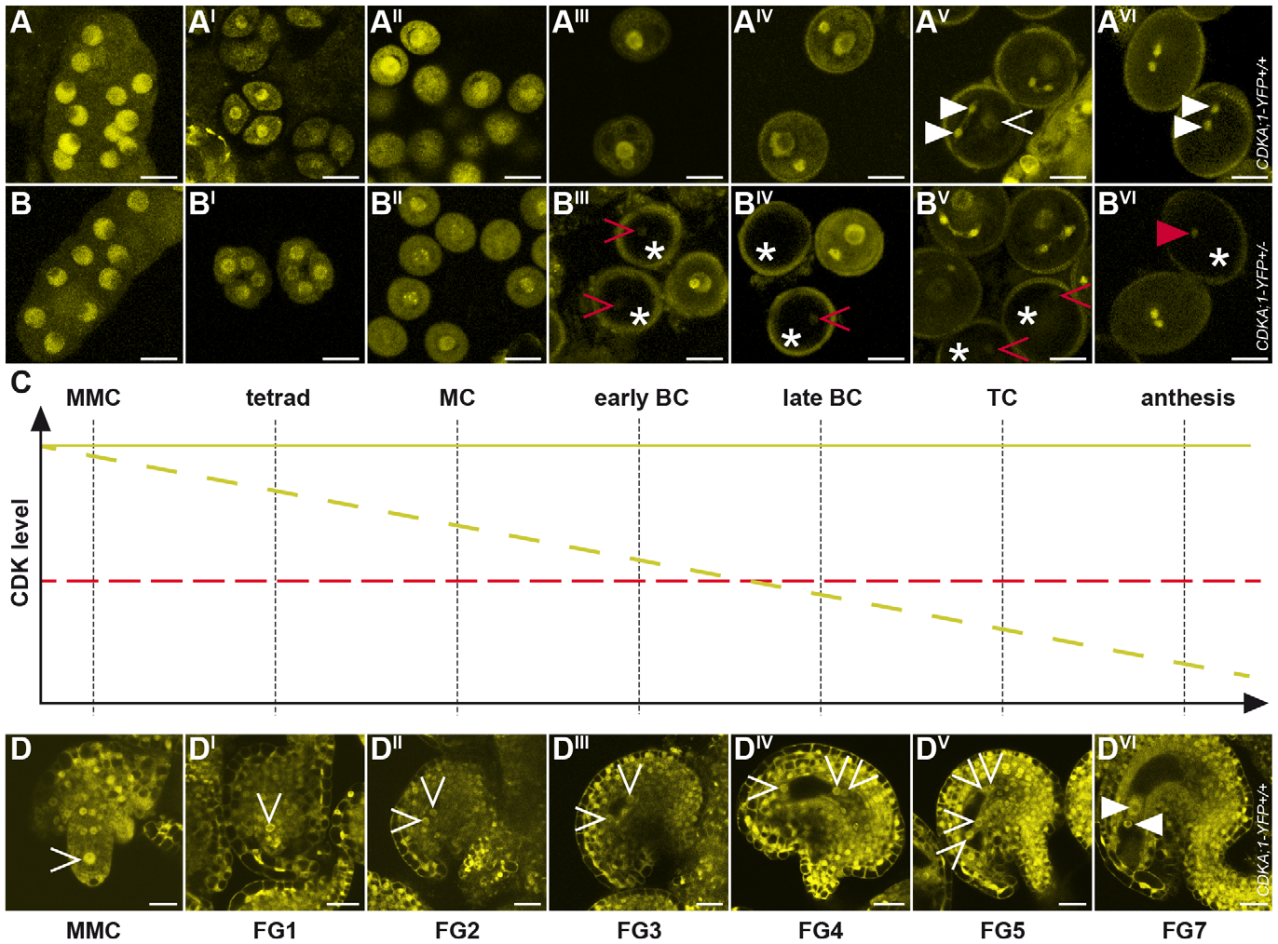
Accumulation and localization of CDKA;1-YFP fusion protein during female and male gametophyte development. (A-A^VI^) Expression of a *PRO_CDKA;1_:CDKA;1-YFP* construct completely rescues *cdka;1* pollen resulting in wild-type-like pollen with one vegetative cell (arrowhead in A^V^) and two sperm cells (triangle in A^V^ and A^VI^, see also Figure 1). *CDKA;1* is expressed throughout male gametophyte development but becomes restricted at anthesis to the two sperm cells as revealed by YFP accumulation. (B-B^VI^) A hemizygous *PRO_CDKA;1_:CDKA;1-YFP^+/−^* allele in homozygous mutant *cdka;1^−/−^* plants mimics heterozygous *cdka;1^+/−^* mutant plants that produce pollen of which half resembles wild-type pollen but half comprises one vegetative cell and only one instead of two sperm cells at anthesis (see Figure 1). Continuous observation of the CDK-YFP fusion protein during male gametophyte development showed that the CDKA;1 protein concentration gradually decreased in 50% of pollen (marked by an asterisk) and that around the bicellular stage, clearly one pollen population can be identified that shows no or very little YFP fluorescence; occasionally, residual CDKA;1-YFP protein could be detected in the single sperm pollen at anthesis (red arrowhead in B^VI^). A and B, Microspore mother cell; A^I^ and B^I^, tetrads; A^II^ and B^II^, monocellular stage; A^III^ and B^III^, early bicellular stage; A^IV^ and B^IV^, late bicellular stage; A^V^ and B^V^, tricellular stage; A^VI^ and B^VI^, anthesis. (C) Cartoon summarizing the decrease in CDKA;1 concentration as seen in B-B^VI^. At the bicellular stage, CDKA;1 levels in mutant pollen (dashed yellow line) drop below an assumed threshold (dashed red line) for executing mitosis. Consistent with the disappearance of the YFP fluorescence at this stage, *cdka;1* mutant pollen typically arrest before PMII. (D-D^VI^) CDKA;1-YFP signal appears in all nuclei at all developmental stages of the developing embryo sac (MMC to FG7, arrowheads mark the nuclei in the embryo sac). At maturity, YFP fluorescence is only present in the gametic cells, the egg cell and the central cell (marked by a white triangle in D^VI^). MMC, megaspore mother cell/microspore mother cell; MC, monocellular; BC, bicellular; TC, tricellular; FG, female gametophyte stage.

To determine the localization pattern of CDKA;1, we analyzed the accumulation of a CDKA;1-YFP fusion protein during the development of the female and male germlines (Figure 2A–2A^VI^). Previously, the production of the CDKA;1-YFP fusion protein from the endogenous *CDKA;1* promoter had been found to completely rescue the *cdka;1^−/−^* mutants [29]. On the male side, CDKA;1-YFP occurred in the microspore mother cell (Figure 2A) and, subsequently, in the nucleus of the single-celled microspore (Figure 2A^I^, 2A^II^). After PMI, CDKA;1-YFP was present in both the vegetative and generative cells (Figure 2A^III^, 2A^IV^). Besides in the nucleus of the vegetative cells, CDKA;1-YFP accumulated also in the two sperm cells after PMII (Figure 2A^V^). At anthesis, CDKA;1-YFP could no longer be detected in the vegetative nucleus and the two sperm cells became exclusively marked by the fusion protein, consistent with a terminal state in the vegetative cell and the observation that the two sperm cells are still in S phase in mature pollen grains (Figure 2A^VI^) [30].

Analysis of the female gametophyte development revealed an overall similar accumulation pattern of CDKA;1-YFP. From meiosis onward, CDKA;1-YFP was found in all nuclei at all developmental stages of the developing embryo sac (Figure 2D–2D^VI^). At maturity, CDKA;1-YFP activity withdrew from the accessory cells and was present only in the gametic cells, i.e. the egg cell and the central cell (Figure 2D^VI^).

As this expression pattern is consistent with a function of CDKA;1 throughout the female and male gametogenesis, we analyzed plants that were homozygous for the *cdka;1* mutation and carried a single allele of the *CDKA;1-YFP* rescue construct. This situation mimicked heterozygous mutants in which approximately half of the pollen was arrested at PMII [29]. CDKA;1-YFP could be detected in all meiocytes and in all single-celled microspores (Figure 2B–2B^II^). However, shortly before PMII, the YFP signal diminished in approximately half of the pollen and was not, or only hardly, visible at anthesis in almost 50% of the pollen (Figure 2B^III^–2B^VI^).

Taken together, these observations are consistent with a carry over of CDKA;1 mRNA and/or protein from maternal, i.e. premeiotic and meiotic stages, and a subsequent reduction of CDKA;1 levels during male gametophyte development falling below the S-phase threshold concentration around the second mitotic division in *cdka;1* mutant pollen (Figure 2C). In contrast, almost all embryo sacs were CDKA;1-YFP positive (data not shown), indicating a higher level of maternal *CDKA;1* mRNA and/or protein inheritance at least partially accounting for the absence of a mutant phenotype during female gametogenesis.

### Expression of dominant negative *CDKA;1* versions rescues the *cdka;1* pollen phenotype

To unravel the function of CDKA;1 in early female and male gametogenesis, we assessed the possibility to additionally deplete CDKA;1 function in a heterozygous *cdka;1* mutant background. Recently, the *cdka;1* mutant pollen that is bicellular at anthesis, has been shown to still undergo a second division [28]. However, whereas the egg cell could still fuse with one of the generated sperm cells, karyogamy of the second sperm with the central cell failed for yet unknown reasons. Thus, the transmission rate of the mutant *cdka;1* allele can be severely distorted and is not necessarily a good measure of the primary division activity and we therefore focused in the following analyses only on the pollen phenotypes.

First, we generated artificial micro RNAs (amiRNA) against CDKA;1 (*amiCDKA;1*) and expressed these *amiCDKA;1* constructs in a heterozygous *cdka;1^+/−^* mutant background under the native *CDKA;1* promoter. Indeed, the *cdka;1* mutant phenotype was enhanced in these plants with 14% more bicellular pollen (57%) than in *cdka;1^+/−^* heterozygous plants (43%) and concomitantly, CDKA;1 protein levels were reduced in these plants (Figure 1B, 1D; Figure S1; Table 1). The observed phenotypic enhancement was consistent with inheritance of the CDKA;1 mRNA/protein, but the effect was small. When the *amiCDKA;1* constructs were expressed in a wild-type background, CDKA;1 protein levels could be reduced to approximately the level of the heterozygous plants (Figure S1) but only 5% of the pollen showed a *cdka;1* mutant phenotype (Table 1).

**Table 1:** Pollen phenotypes.

Genotype	Tricellular pollen(%)	Bicellular pollen(%)	Monocellular pollen(%)	n
Col 0	97.8	2.2	0.0	1058
cdka;1+/−	56.8	43.2	0.0	862
fbl17+/−	57.3	42.7	0.0	936
cdka;1+/− krp1−/−	68.8	31.2	0.0	841
cdka;1+/− krp2−/−	55.4	44.6	0.0	1141
cdka;1+/− krp3−/−	57.0	43.0	0.0	1167
cdka;1+/− krp4−/−	56.4	43.6	0.0	1093
cdka;1+/− krp5−/−	54.0	46.0	0.0	1108
cdka;1+/− krp6−/−	70.4	29.6	0.0	798
cdka;1+/− krp7−/−	56.0	43.9	0.0	1370
cdka;1+/− D146N#1	90.3	9.7	0.0	704
cdka;1+/− D146N#2	91.0	9.0	0.0	762
cdka;1+/− D146N#3	89.9	10.1	0.0	690
cdka;1+/− K33R#1	85.3	14.7	0.0	938
cdka;1+/− K33R#2	93.2	6.8	0.0	999
cdka;1+/− K33R#3	87.7	12.3	0.0	839
cdka;1+/− Pstaire-dead#1	56.7	43.3	0.0	895
cdka;1+/− Pstaire-dead#2	55.3	44.7	0.0	857
cdka;1+/− Pstaire-dead#3	56.0	44.0	0.0	923
cdka;1+/− amiCDKA;1	42.6	57.4	0.0	1098
amiCDKA;1	95.3	4.7	0.0	1930
cdka;1+/− PRO_UBQ_:FBL17	78.3	21.7	0.0	1277
cdka;1+/− PRO_CDKA;1_:FBL17	72.5	27.5	0.0	668
fbl17+/− krp1−/−	64.6	35.4	0.0	1391
fbl17+/− krp2−/−	55.8	44.2	0.0	976
fbl17+/− krp3/−	72.6	27.4	0.0	1258
fbl17+/− krp4−/−	63.5	36.5	0.0	1011
fbl17+/−krp5−/−	56.4	43.6	0.0	890
fbl17+/− krp6−/−	66.2	33.8	0.0	1241
fbl17+/− krp7−/−	67.9	32.1	0.0	913
fbl17+/− rbr+/−	69.8	30.2	0.0	742
fbl17+/− D146N	79.6	20.4	0.0	781
fbl17+/− e2fa−/−	63.6	11.3	25.2	969
cdka;1+/− fbl17+/−	54.1	36.9	9.0	842

Pollen from anthers just before flowering of wild type and the indicated genotypes was stained with DAPI and epifluorescence was observed under UV illumination. n = total number of pollen analyzed.

Next, we generated plants that produced a *CDKA;1* mutant version in which Asp146 was replaced by Asn (*CDKA;1^D146N^*) driven by the *CDKA;1* promoter. In mammalian and yeast kinases, homologous substitutions are known to abolish ATP access to the catalytic cleft, while cyclins and substrates are still bound, thus functioning as dominant-negative proteins [31]. Like in mammals and yeast, previous studies in plants have shown that this substitution has no kinase activity [32]. Surprisingly, the expression of *CDKA;1^D146N^* in heterozygous *cdka;1^+/−^* mutants partially rescued the pollen phenotype, namely between 85% and 93% of the pollen were tricellular and only 7% to 15% bicellular versus the typical 57% tricellular/43% bicellular pollen in heterozygous *cdka;1^+/−^* plants (Figure 1B, 1D; Table 1). Similarly, a *StrepIII-CDKA;1^D146N^* version also partially rescued the *cdka;1* pollen phenotype (data not shown).

To test whether this effect was limited to the *CDKA;1^D146N^* construct, we generated two additional transgenic plants, another dominant-negative allele *CDKA;1^K33R^* fused to a StrepIII-tag and one *CDKA;1^PSTAIRE-dead^* version in which the archetypically conserved PSTAIRE domain in the C-helix of the N-terminal cyclin-binding domain (residues Glu42–Glu57) and neighboring residues (from Gly43–Lys56) had been replaced by 14 alanines (designated PSTAIRE-dead), resulting in a protein that could presumably not bind to cyclins any longer. Similarly to *CDKA;1^D146N^*, *CDKA;1^K33R^* could partially rescue the pollen development of *cdka;1^+/−^* heterozygous mutants (Table 1). In contrast, *cdka;1^+/−^* mutants expressing the *CDKA;1^PSTAIRE-dead^* construct showed the typical *cdka;1* pollen arrest at anthesis (Table 1). Thus, the PSTAIRE domain and, probably, the binding to the cyclin partner are important for the observed effect of the dominant-negative protein version. We conclude that, in contrast to the expected titration of cyclins or the blocking of the phosphorylation of substrates required for cell-cycle progression, the expression of the dominant-negative *CDKA;1* versions from the *CDKA;1* promoter interfered with negative factors of the cell cycle.

### Mutants in KRP-type CDK inhibitors can rescue *cdka;1* and *fbl17* mutant pollen

**Figure 3:**
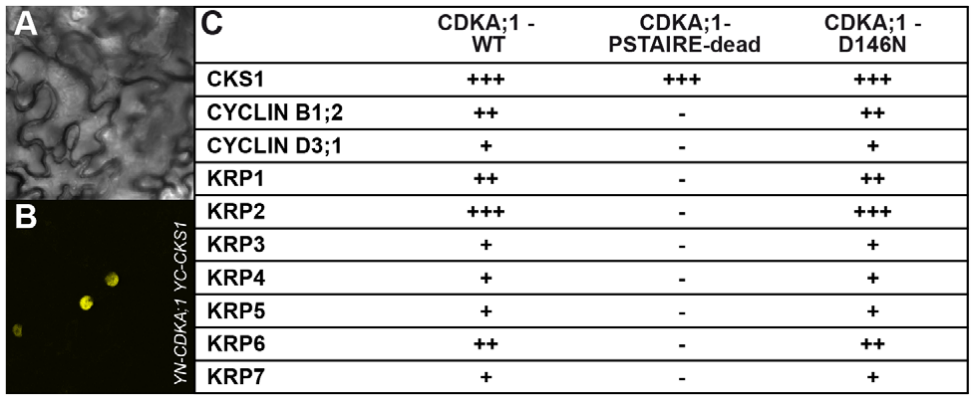
Interaction assays of dominant negative versus wild-type CDKA;1 variants. Positive BiFC assays showing the interaction of CDKA;1 with KRP1 under bright field (A) and epifluorescence (B). (C) The dominant-negative CDKA;1 variant CDKA;1^D146N^ interacting with both positive (cyclins) and negative (KRPs) regulators similar to the wild-type CDKA;1 form. The CDKA;1^PSTAIRE-dead^ variant shows neither interaction with cyclins nor with KRPs, but can still bind to the cofactor CKS. The interactions are presented in a semiquantitative manner based on the number of positive cells obtained and the observed fluorescent intensities. All interactions were at least three times independently tested.

Important negative regulators of plant cell-cycle progression are CDK inhibitors of the KRP class [15], [16]. Moreover, *KRP6* and *KRP7* had been found previously to be expressed during male gametogenesis and overaccumulation of these inhibitors to be associated with cell-cycle arrest during pollen development [23]–[25]. Indeed, *CDKA;1^D146N^* could bind not only to cyclins but also to KRPs in bimolecular fluorescence complementation (BiFC) assays, whereas *CDKA;1^PSTAIRE-dead^* bound only to a cyclin-dependent kinase subunit (CKS) (Figure 3A, 3B, 3C).

The results suggested that the stoichiometric ratio between CDK inhibitors and CDKA;1 is crucial for the formation of two sperm cells. Therefore, we isolated null mutants in *KRP3* and *KRP4* (Figure S2A, S2B) and combined them as well as the previously described mutants for *KRP1*, *KRP2*, *KRP5*, *KRP6* and *KRP7* [23], [33], [34] with *cdka;1^+/−^* mutants. The *krp6^−/−^* mutants and, to a lesser extent *krp1^−/−^*, could rescue the *cdka;1* mutant pollen phenotype (Table 1).

These results raised the question how cell-cycle progression is driven in the absence of CDKA;1. Therefore, homozygous *cdka;1^−/−^* mutants complemented with only a hemizygous CDKA;1-YFP rescue construct were reassessed (see above). We found that mutant pollen had occasionally only one single sperm-cell-like cell that still contained residual levels of the CDKA;1-YFP fusion protein (Figure 2B^VI^). Thus, the observed rescue of *cdka;1* by the *krp1* and *krp6* mutants is, at least partially, due to a liberation of remaining CDKA;1 levels in mutant pollen, additionally implying a previously not recognized role for *KRP1* during gametogenesis.

**Figure 4:**
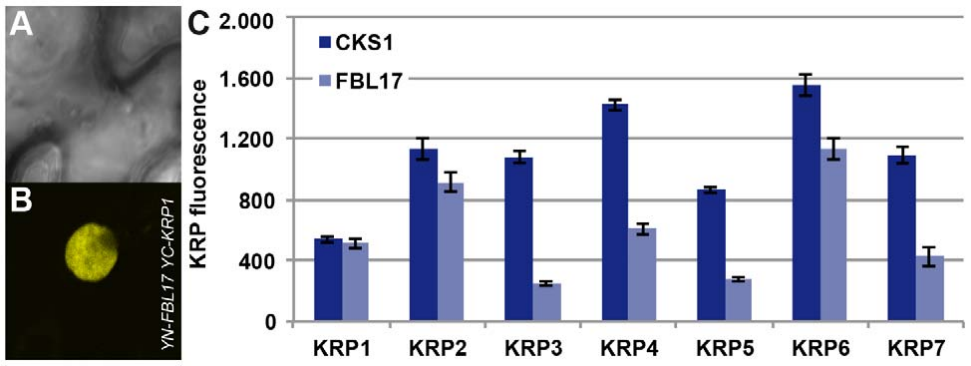
Interaction of FBL17 with KRPs in BiFC assays and degradation promotion of KRP *in vivo*. Two nonfluorescent fragments (YN and YC) of the yellow fluorescent protein (YFP) fused to FBL17 (YN-FBL17) and seven different CDK inhibitors (YC-KRP1–7). Co-production of FBL17 and KRP1-KRP7 fusions in tobacco leaves reconstituted the expected yellow fluorescence for all seven protein combinations. Exemplarily, one interaction in the BiFC assays is shown in (A–B) displaying the interaction of FBL17 with KRP1 under bright field (A) and epifluorescence (B). (C) Transient expression assays were conducted in tobacco leaves to determine whether FBL17 can target CDK inhibitors for degradation. Among the seven KRPs, FBL17 can especially reduce the fluorescence, implying protein degradation, for KRP3, KRP4, KRP5 and KRP7, whereas the fluorescence intensity of KRP2 and KRP6 diminished only moderately after co-infiltration with FBL17. CKS1 was used as a reference.

As FBL17 had been found to control KRP6 and KRP7 levels during male gametogenesis [23], [24], we asked whether KRP1, as well as the other KRPs, might be also controlled by FBL17. Therefore we combined mutants in all 7 *KRPs* with *fbl17^+/−^* mutant plants. In addition to the previously reported partial rescue of *fbl17^+/−^* by *krp6^+/−^* mutants [23], we found that mutants in *krp1*, *krp3*, *krp4* and *krp7* could partially rescue *fbl17^+/−^*, as seen by an increased proportion of tricellular pollen at anthesis in comparison to heterozygous *fbl17^+/−^* plants (Table 1). To further test a possible role of FBL17 in degrading other KRPs than KRP6 and KRP7, we co-expressed FBL17 with YFP fusion proteins of all seven KRPs in tobacco leaf cells and monitored the fluorescence intensity in comparison with the co-expression of KRP-YFP fusion proteins and CKS1 (Figure 4A, 4B, 4C). In this assay, co-production of FBL17 reduced the fluorescence intensity especially of KRP3, KRP4, KRP5 and KRP7, and to a lesser extent of KRP2 and KRP6. This finding is consistent with the mediation of the proteasomal degradation of all or almost all KRPs through FBL17. In further support of this conclusion, we found that, after *CDKA;1^D146N^* had been combined with *fbl17^+/−^* mutants, the proportion of wild-type-like tricellular pollen increased presumably due to a titration of overaccumulating levels of KRPs and, thus, at least in part, to a liberation of CDKA;1 (Table 1).

### E2F and RBR1 directly control *FBL17* expression

**Figure 5:**
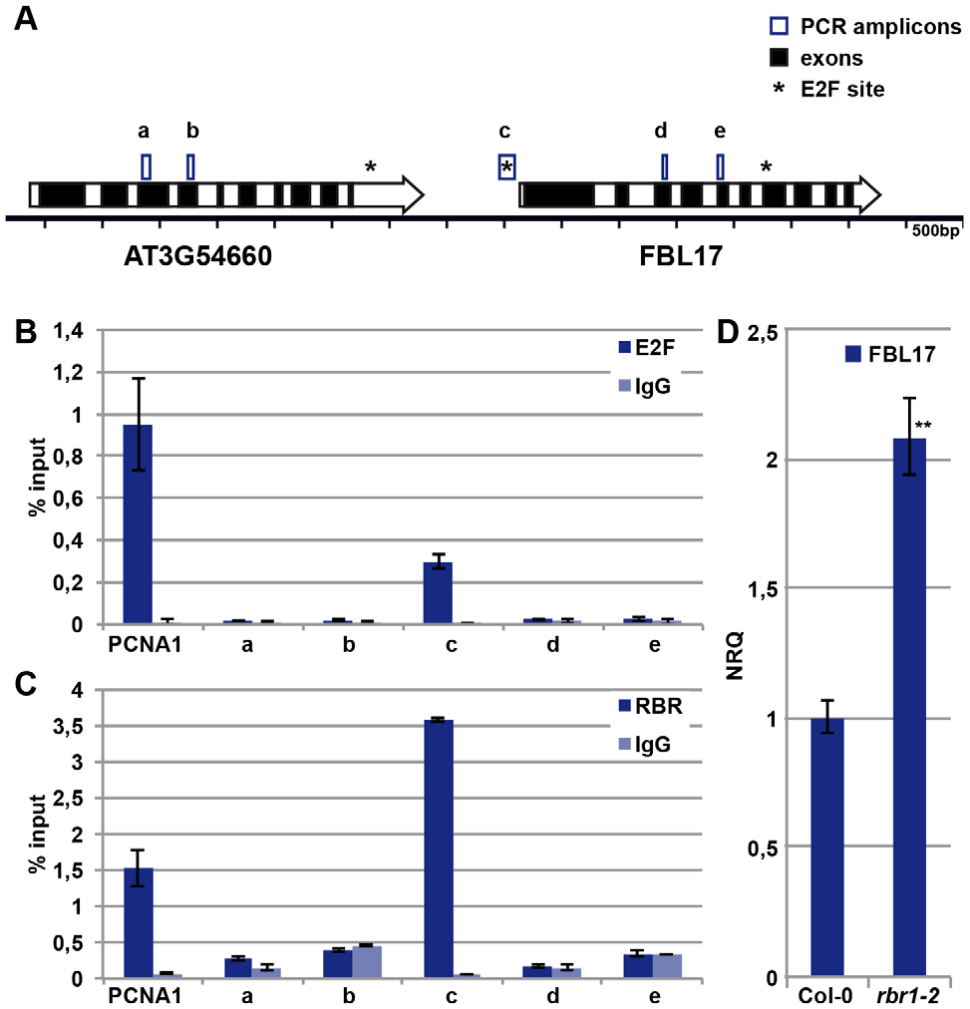
E2FA and RBR control the expression of *FBL17*. (A) Sketch of the genomic region of *FBL17* in the *Arabidopsis* genome. Genes are represented by large arrows with exons in black and introns in white, predicted E2F binding sites are marked with an asterisk, and the five PCR amplicons used in ChIP analyses are boxed (a, b, c, d, e). (B) E2FA ChIP. Wild-type plants were used for ChIP experiments with an antibody directed against E2FA. The amplicon ‘c’ in the promoter region of *FBL17* showed enrichment for E2FA, whereas the regions ‘a’, ‘b’, ‘d’ and ‘e’ were not specifically amplified. The known E2FA target, *PCNA1*, was used as positive control. Negative controls were done without antibody (IgG). (C) RBR1 ChIP. Transgenetic plants expressing *PRO_RBR1_:RBR1-mRFP* were used in ChIP with a DsRed antibody. The amplicon ‘c’ in the promoter region of *FBL17* showed enrichment for RBR1-mRFP, in contrast to the regions ‘a’, ‘b’, ‘d’ and ‘e’. The known RBR1 target, *PCNA1*, was used as positive control. Negative controls were done without antibody (IgG). (D) Quantitative expression analysis of *FBL17* by qRT-PCR in the wild type and *rbr1–2^−/−^* mutants. The mean plus/minus standard deviation of the normalized relative quantities (NRQ) of three biological replicates are shown. The stars indicate statistically significant differences based on a t-test of log-transformed data with a p<0.01.

Given its central importance, we next asked how FBL17 is regulated. The promoter of *FBL17* contains a putative binding site for the transcription factor E2F [35], and *FBL17* transcripts strongly accumulate in plants that co-overexpress the transcription factor E2FA (also designated E2F3) along with its dimerization partner DPa [23]. In support of a direct regulation by E2F, we could amplify the promoter fragment that contains the predicted E2F binding site after chromatin immunoprecipiation (ChIP) with an antibody against E2FA (Figure 5A, 5B). Fragments further upstream and downstream of the putative E2F binding site could not be amplified, while a strong enrichment for a promoter fragment of the known E2F target *PROLIFERATING CELL NUCLEAR ANTIGEN 1* (*PCNA1*) as a positive control was obtained (Figure 5B).

The action of E2F is counterbalanced by Rb in mammalian cells [36], [37], and to test whether *FBL17* is a direct target of the Rb homolog RBR1 in plants, we performed ChIP experiments with RBR1 using plants that express a functional RBR1-mRFP fusion protein [38]. As a positive control, RBR1 binding to the known RBR1 target PCNA1 was observed, while RBR1 ChIP did not precipitate heterochromatic loci used as negative controls (Figure 5A, 5C; data not shown). As a proof of direct binding of RBR1 to the *FBL17* promoter, we amplified one fragment of the *FBL17* promoter from the precipitated DNA of the RBR1-mRFP-producing plant (Figure 5A, 5C). Notably, this fragment contained the predicted E2F binding site (Figure 5A) and was the same fragment that could be precipitated in the E2F ChIP experiments (Figure 5B). In contrast, fragments further upstream and downstream of the putative E2F site could not be amplified after ChIP suggesting that the interplay between E2F and RBR1 regulates *FBL17* expression. Furthermore, the *FBL17* transcript level was significantly upregulated in *rbr1–2* ^−/−^ mutants (Figure 5D).

To check then the biological importance of this wiring, we generated the *fbl17^+/−^ rbr1^+/−^* double mutant. The mutant pollen phenotype of *fbl17^+/−^* plants was partially rescued, i.e. 43% bicellular pollen in *fbl17^+/−^* versus 30% in *fbl17^+/−^ rbr1^+/−^* (Table 1). This is consistent with the higher level of *FBL17* expression observed in *rbr1* ^−/−^ mutants (Figure 5D) and, although the *fbl17* allele used here is a transcriptional null allele [23], a modulation of FBL17 activity appears to be possible, assuming that similar to *CDKA;1*, *FBL17* mRNA and/or protein is carried over from meiotic stages.

Conversely, we hypothesized that mutants in *e2fa* should enhance the *fbl17* mutant phenotype. Analysis of embryo sac development in *e2fa^−/−^ fbl17^+/−^* mutants did not reveal any deviation from the wild type, but a new class of mutant pollen was found that consisted of only one single cell versus the tricellular composition of pollen at anthesis in the wild type (Figure 1E; Table 1).

Thus, the direct control of E2FA and RBR1 is decisive for FBL17 action at least during male gametophyte development. Moreover, the new phenotype of the *e2fa^−/−^ fbl17^+/−^* mutants demonstrates that FBL17 already functions in the first division cycle of pollen development and likely, similar to the situation in *cdka;1^+/−^* mutants, is masked by a maternal carry over of *FBL17* transcript and/or protein.

### Concomitant loss of CDKA;1 and FBL17 results in unicellular female and male gametophytes

The early function of FBL17 reinforced the idea that the interplay between CDKA;1 and FBL17 also controls PMI. Therefore, we combined the *cdka;1* and *fbl17* mutants that are linked by 3 cM on chromosome 3. As no double heterozygous mutants could be recovered in the progeny of a backcross of *cdka;1^+/−^ fbl17^+/−^* with the wild type, we used the above-described *CDKA;1-YFP* rescue line in *cdka;1^+/−^* for combinations with *fbl17^+/−^*. We could stably generate plants in which mutations in *CDKA;1* and *FBL17* were *in cis* located in the presence of a heterozygous *CDKA;1-YFP* rescue construct *in trans*. In these plants, approximately 25% of the gametophytes were expected to be devoid of both *FBL17* and *CDKA;1* (either the endogenous transcripts or the *CDKA;1-YFP* rescue construct). However, due to the identified inheritance of CDKA;1 and probably of FBL17 as well, a mutant phenotype might be underrepresented. Analysis of pollen of these double mutants revealed that in nearly 10% the pollen contained only one single cell (Figure 1C, 1E, Table 1). Quantification of the fluorescence intensity of 4′,6-diamidino-2-phenylindole (DAPI)-stained mutant versus wild-type pollen showed that the single-celled pollen had a DNA content of 1C, i.e. arrested in G1 (Figure 1F). Thus, CDKA;1 together with FBL17 control S-phase entry during both the first and second mitotic division during male gametogenesis.

**Figure 6:**
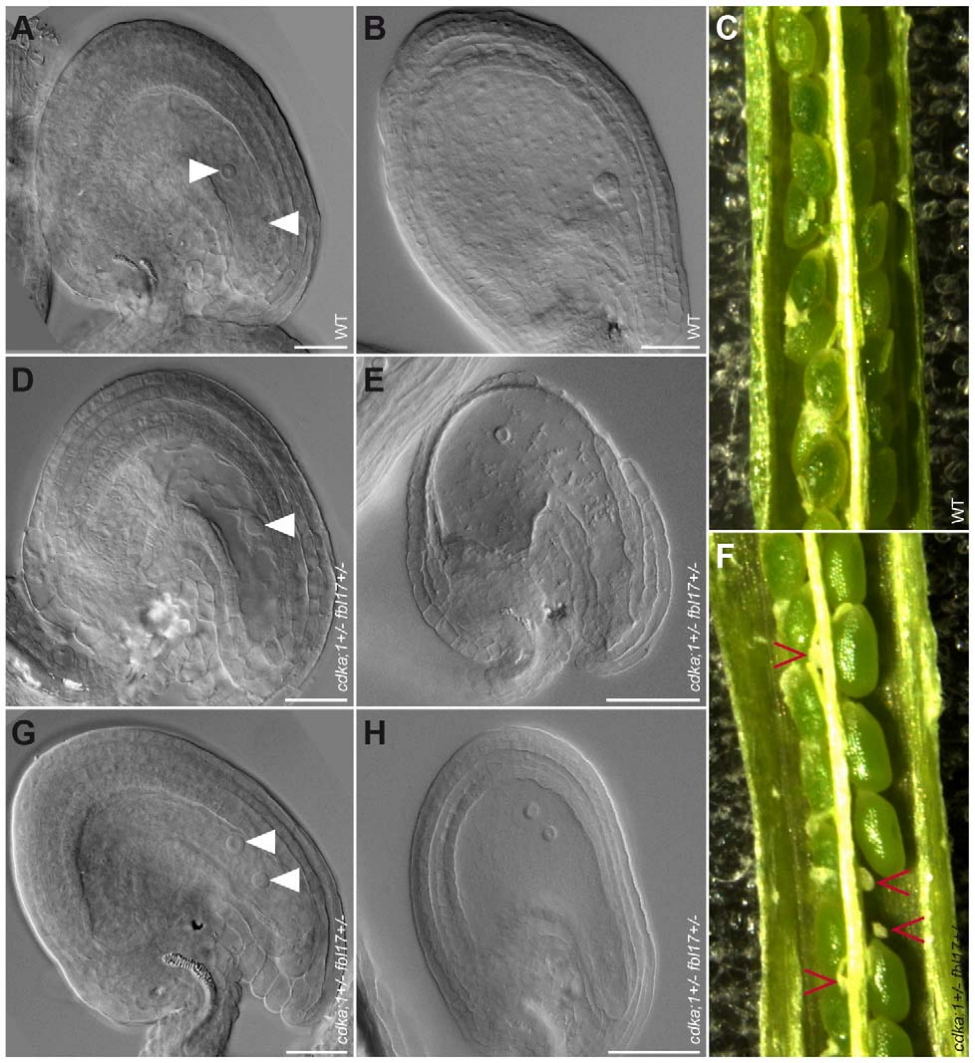
Mature ovules and seed development in wild type and *cdka;1 fbl17* double mutant. (A–C) Wild-type embryo sac and seed development; (D–F) aberrant development in a class of *cdka;1 fbl17* mutant plants. (A) Wild type showing a typical cellular morphology (arrowheads pointing to the central cell nucleus and the egg cell nucleus from top to bottom, respectively). Aberrant morphologies in the *cdka;1^+/−^ fbl17^+/−^* double mutant with one (D), or two (G) nuclei in the absence of a cellularized egg apparatus. (B) While the wild-type seed 3 days after pollination has a normal embryo and endosperm development, the double mutant seed development collapsed after pollination, with only one (E) or two (H) nuclei staying in the middle of the empty embryo sac. (C) Wild type showing normal seed development 6 days after pollination, while *cdka;1^+/−^ fbl17^+/−^* double mutants show approximately 24% seed abortion (F) after pollination with the wild-type pollen.

Next, we analyzed the female gametophyte in *cdka;1^+/−^ fbl17^+/−^ ProCDKA;1:CDKA;1-YFP^+/−^* plants. Approximately 25% of embryo sacs in these plants had a different developmental pattern than in the wild type. At maturity, embryo sacs contained only one or two nuclei of similar size and presented no sign of cellularization (Figure 6A, 6D, 6G). The mutant embryo sacs remained unfertilized after pollination with wild-type pollen (Figure 6B, 6E, 6H), indicating that, as expected, they were not functional. Consistently, we found aborting ovules in differentiating siliques (Figure 6C, 6F). Notably, in the absence of the *CDKA;1-YFP* rescue construct, *cdka;1* together with the *fbl17* mutant allele was never transmitted through either the female or the male parent (data not shown), implying that the combination of both genes is essential for the development of the two gametophytes. Taken together, CDKA;1 and FBL17 also control the first and second mitotic division cycle during embryo sac formation.

### Modeling the G1/S module

**Figure 7:**
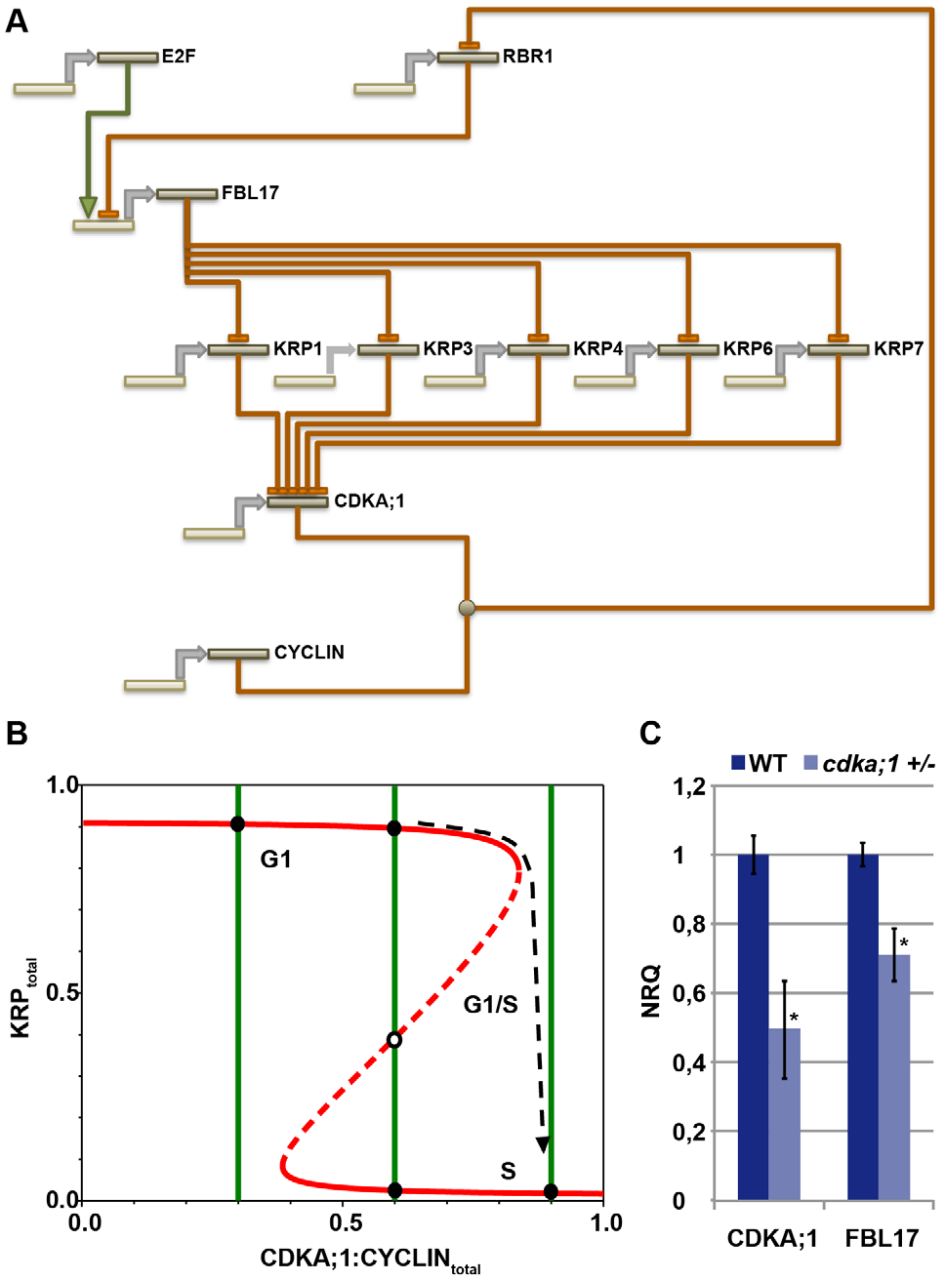
General G1/S phase cell-cycle control module. (A) The transcription factor E2F activates the expression of *FBL17*, which is repressed by RBR1. FBL17 targets the CDKA;1 inhibitors KRP1, KRP3, KRP4, KRP6 and KRP7 for proteasome-dependent degradation, enabling the germ cell to progress through S phase. Phosphorylation of RBR by the CDKA;1-cyclin complex will relieve the inhibition of the S-phase genes and allows transcription of the *FBL17* gene. Promoters and genes are depicted in light sand color; proteins in dark sand; transcription is indicated by a grey arrow; negative regulation, i.e. at the transcriptional or protein level, is shown by rust-colored lines with a blunt end; positive regulation by a green line with a green arrowhead. Receiving input is placed above and executing output under the respective gene/protein. (B) The model presented in A gives rise to a bistable switch controlling the G1-to-S transition in the plant cell cycle. KRPs inhibit the CDKA;1-cyclin complexes, which in turn downregulate the levels of KRPs by phosphorylating and inhibiting RBR1, thereby activating E2F-dependent FBL17 synthesis leading to the degradation of KRPs. The antagonistic interaction between CDKA;1 and KRP is illustrated by the two curves (red and green for KRP and for CDKA;1, respectively) along which the rates of synthesis and degradation of KRPs and the CDKA;1-cyclin complexes are exactly balanced. The KRP balance curve has an inverse S-shape with high and low levels, depending on the CDKA;1-cyclin values. The dashed branch of the balance curve represents unstable steady states. At low CDKA;1-cyclin levels, only one steady state exists with high KRP levels and low CDKA;1 activity. At intermediate CDKA;1 levels, the system is bistable with three steady states. At high CDKA;1-cyclin values, the steady-state level of KRP is low and the CDKA;1-cyclin complexes are fully active. The transition from high to low KRP values corresponds to the G1-to-S transition. (C) Quantitative expression analyses of *CDKA;1* and *FBL17* in wild type and heterozygous *cdka;1* mutants. The mean plus standard deviation of the normalized relative quantities (NRQ) of three biological replicates are shown. The stars indicate statistically significant differences based on a t-test of log-transformed data with a p<0.05. As expected, the expression of *CDKA;1* drops by approximately 50% in the mutant. In addition, *FBL17* expression declines, consistent with a prediction of the model presented in A and B.

The data presented above show that the CDKA;1-KRPs-FBL17-RBR-E2F pathway builds a general G1/S module that controls entry into S phase in *Arabidopsis*. This module involves four steps of negative regulation, i.e. RBR1 repressing *FBL17* (this study), FBL17 mediating the degradation of KRPs (this study and [23], [24]), KRPs inhibiting CDKA;1 [15], [16], [39], and CDKA;1 phosphorylating RBR1 and inhibiting it [12] (Figure 7A). Biomathematical simulations of this G1/S module revealed that this wiring gives rise to stable and self-maintaining steady states with a pronounced hysteresis (Figure 7B, Dataset S1). Moreover, the decision whether to move from G1 into S phase strongly depended on the concentrations of KRPs, consistent with the experiment with dominant-negative *CDKA;1* variants.

This model also predicted that the expression of *FBL17* should intensely rely on CDKA;1-cyclin activity levels. In a *cdka;1* mutant background, the system not only runs out of kinase to promote S-phase entry, but the remaining kinase activity might also be inhibited, because less *FBL17* might be expressed due to the reduced repression of *RBR1*, and thus the abundance of KRPs increases. To test this assumption, we introgressed a previously generated promoter reporter line for *FBL17* into *cdka;1* heterozygous mutants [23]. However, GUS levels could not reliably be quantified. Thus, we next performed qRT expression analyses from anthers of the fifth and fourth floral buds before the first flower opens, since under our growth conditions anthers of these flowers contain monocellular and bicellular pollen and thus, there was no bias for the number of pollen nuclei between wild-type and mutant plants. Consistent with the prediction of our model, we observed a significant (unpaired t-test, p<0.05) reduction in *FBL17* expression at both floral stages of heterozygous *cdka;1* mutants (Figure 7C, data not shown). Next, we functionally tested this feedback wiring by uncoupling the expression of *FBL17* from its regulation by RBR1. Indeed, expression of *FBL17* from the ubiquitin as well as the *CDKA;1* promoter in a heterozygous *cdka;1* mutant situation could partially rescue the pollen phenotype (Table 1). Thus, we conclude that the proposed mathematical model captures central aspects of the presented G1/S module.

## Discussion

Here, we have shown that cell-cycle progression during female and male gametogenesis is under the control of a common S-phase module comprising the transcriptional regulators E2F and RBR1, the F-box protein FBL17, CDK inhibitors of the KRP class, and CDKA;1. This G1/S module provides a molecular-genetic framework of cell-cycle control that probably functions in sporophytic parts of *Arabidopsis* and possibly of other plants as well.

### Toward the mechanism of CDK inhibition by KRPs

At the heart of the here-identified G1/S cell-cycle phase regulatory module is the inhibition of CDKA;1 by KRPs. In *Arabidopsis*, the *KRP* genes form a gene family of seven members with seemingly highly overlapping functions as suggested by the absence of an obvious mutant phenotype in single *krp* mutants [33], [34]. Typically, null mutants for components that function in one regulatory pathway, e.g. here for *cdka;1*, *fbl17* and several *krps*, show an epistatic relationship. However, due to the high level of carry over that we identified here for CDKA;1, but which is likely true for many other cell-cycle as well as developmental regulators, genetic interactions in one pathway, e.g. dosage sensitivity of CDKA;1 toward KRPs, could be revealed. Therefore, the heterozygous *cdka;1^+/−^* mutant background, with gradually decreasing levels of maternally provided CDKA;1, offered a unique opportunity to quantitatively dissect the regulatory cascades at the S-phase entry and to evaluate the effects of the redundantly acting KRPs.

The CDK dosage sensitivity became especially apparent when dominant-negative CDK variants were expressed in a *cdka;1* mutant background. These dominant-negative CDK versions had been used previously in plants, as well as in yeast and metazoans, and are known to be completely devoid of kinase activity [32], [40]–[45]. Instead of the expected negative effect on cell-cycle progression, these kinase versions could partially rescue the pollen phenotype of heterozygous *cdka;1^+/−^* mutants. Our data suggest that moderate expression levels of dominant-negative CDKs first bind to and titrate CDK inhibitors before sufficiently reducing the levels of active CDK-cyclin complexes. Indeed, it has been found that the biologically important concentration of cyclin lies in a nM range in *Xenopus laevis* [46]. Although the cyclin concentrations have not been quantified in *Arabidopsis*, it is well known that *Arabidopsis* cyclin promoters are very strong, implying that the concentrations are high in plants as well [47]–[49]. In contrast, the abundance of KRPs seems to be very low [50]–[52]. Thus, the moderate expression of ‘dominant-negative’ kinases might be a useful tool to titrate CDK inhibitors outside of pollen in other developmental/physiological contexts.

Interestingly, only the mutant CDK variants that could bind to cyclins, i.e. *CDKA;1^D146N^* and *CDKA;1^K33R^*, partially rescued *cdka;1* mutants. In contrast, expression of the *CDKA;1^PSTAIRE-dead^* version, which could bind to the CKS cofactor, but not to cyclins, did not rescue the defective pollen in *cdka;1* mutants, giving rise to the hypothesis that KRPs may preferentially bind to CDK-cyclin complexes or, at least, have a higher affinity for the dimer than for the individual monomers, consistent with previous interaction assays showing that monomeric CDKs and cyclin were hardly targeted *in vitro* by KRPs in contrast to a CDK-cyclin complex [39]. Revisiting previously published yeast two-hybrid (Y2H) interaction data of ICK1/KRP1 with CDKA;1 and D-type CYCLIN 3;1 (CYCD3;1) also revealed that deletion of the presumptive CDK binding site strongly reduced the interaction of ICK1/KRP1 with CYCD3;1, further supporting the assumption that KRPs preferentially target a CDK-cyclin dimer [39], [53].

The CDK inhibitor p27^Kip1^ from mammals belongs to the class of intrinsically unstructured, also called intrinsically disordered or natively unfolded, proteins [54], [55]. Kinetic analyses of p27^Kip1^ have suggested that its folding is induced through binding to the cyclin partner and then reaches the Cdk partner. Consequently, p27^Kip1^ has the highest affinity for the Cdk-cyclin complex, followed by a preference for the cyclin partner over the isolated Cdk [56]. Thus, it is tempting to speculate that KRPs from plants have very similar structural and kinetic properties, especially because in the above-mentioned Y2H experiments the interaction of ICK1/KRP1 was much stronger with CYCD3;1 than with CDKA;1, although it is unclear whether *CYCD3;1* and *CDKA;1* are equally well expressed in the yeast assay [53].

Previously, a *cdka;1* mutant version, in which a conserved threonine (T161) in the T-loop that needs to be phosphorylated for full CDK activity was exchanged with a nonphosphorylatable alanine or valine (CDKA;1^T161A^ and CDKA;1^T161V^), had been reported to be unable to rescue the *cdka;1* mutants [8], [32]. Interestingly, this CDK version could not rescue the *cdka;1* mutant pollen, indicating that CDK inhibitors may only target activated CDK-cyclin complexes. In contrast, mammalian p27^Kip1^ can bind to CDKs irrespective of its T-loop phosphorylation [57], but due to the different positions of the CDK-cyclin-binding domain in p27^Kip1^ versus KRPs, both proteins probably make contact to different parts of CDKs and cyclins. Thus, an important next step in the understanding of the KRP action is the unraveling of their crystal structure when bound to plant CDK-cyclin complexes.

### Conservation and divergence of control mechanisms in G1/S regulatory networks

Because of its importance for growth and development, the S-phase entry has been extensively studied in yeast and metazoans. The crucial aspect of progression into S phase is the activation of the transcription factor E2F that is repressed by Rb in metazoans. Furthermore, Whi5, an analogous transcriptional repressor in yeast, blocks the activity of the S-phase transcriptional regulator SBF [58]. As representatives of the *Plantae* supergroup of the eukaryotic kingdom, plants are much more distantly related to fungi and metazoans, which are both in the same supergroup of *Opisthokonts* [59]; therefore, a comparison of the cell cycle of plants with that of yeast and metazoans will be important to understand the origin and evolution of cell-cycle control in eukaryotes [60]. As the inactivation of the plant homolog RBR1 and, hence, the release of plant E2F homologs are seemingly conserved in plants, early eukaryotes might already have a complex repertoire of cell-cycle control genes [60], [61].

An important mechanism in metazoans and yeast is the positive feedback regulation of E2F/SBF onto its own activity. An initial phosphorylation of Rb or Whi5 reduces the repression of E2F/SBF and promotes the expression of *cyclin E* and *Cln2*, respectively. The increasing levels of these cyclins fully activate the S-phase kinases Cdk2 in metazoans or CDC28p in yeast, resulting in the complete inactivation of Rb/Whi5 [62], [63]. Our data show that the principle of this double negative wiring of Rb proteins is conserved in plants. However, the regulators or their relative importance differ in the plant cell cycle: RBR1 represses E2F that activates *FBL17*, which, in turn, releases the repression of CDKA;1 that can then phosphorylate RBR1, presumably leading to its complete inactivation. Thus, rather than liberating a positive factor, such as a cyclin, plants inactivate another repressor, adding one layer of double negative regulation. Based on our simulations, this wiring can give rise to a strong bistable system with hysteresis. The observation that the concomitant loss of CDKA;1 and FBL17 results in a complete arrest of gametogenesis, underlines the crucial importance of this regulation. Nevertheless, due to the still very limited knowledge about plant cyclins, we cannot exclude that, in addition to the FBL17 loop, another pathway leads to the transcriptional activation of cyclins by E2F in plants.

Conversely, the transcriptional control of the protein degradation machinery, targeting CDK inhibitors, by E2F and Rb (or their functional analogs) appears to be a universal regulatory mechanism. In animals, degradation of the CDK inhibitor p27^Kip1^ is mediated by the F-box protein Skp2, which has been found to be a direct target of E2F and Rb regulation [64]. Perhaps a similar regulation is found in fission yeast (*Schizosaccharomyces pombe*), where the MBF function is executed by the transcriptional regulator Cdc10/Rep2 and its inactivation results in a G1 arrest [65]. Remarkably, this arrest is accompanied by high levels of the Cdk inhibitor Rum1, but it is currently unclear how Rum1 is regulated in this context. A simple hypothesis is that Cdc10 activates the degradation of Rum1, possibly through the transcriptional activation of a yet unknown F-box protein or another component of the protein degradation machinery, ultimately hinting at still unexplored parallels between the plant, animal and yeast cell cycles, and revealing general principles and global constraints of cell-cycle control in eukaryotes.

## Materials and Methods

### Plant material and growth conditions

The *Arabidopsis thaliana* (L.) Heynh. plants were all derived from the Columbia (Col-0) accession. Detailed information on mutant lines used can be found in the extended experimental procedures (Text S1). All genotypes were determined by polymerase chain reaction (PCR) with the primers indicated in Table S1. All seeds were surface-sterilized with chloride gas, sown on 0.8% Phyto agar plates (half-strength Murashige and Skoog (MS) salts and 1% sucrose) and grown under neutral conditions (12 h light at 21°C, and 12 h dark at 17°C). After germination, plants were transferred to soil and grown under long-day conditions (16 h day/8 h night regime at 22°C/18°C). For crosses, flowers of the female parent were emasculated 2 days before anthesis and hand-pollinated 2 days later.

### Constructs and transformation

All manipulations were performed using standard molecular methods, details on the construction of transgenic lines can be found in the extended experimental procedures and Table S1 listing primer sequences.

### Protein work

BiFC was assayed as previously described [66]. Co-injection experiments were performed with KRPs in pEXSG-YFP and CKS1 or FBL17 in pEXSG-CFP. Both Gateway compatible vectors were a kind gift of Marcel Wiermer (AG Romeis, MPIZ, Cologne). Only cells with YFP and CFP signals were chosen for measurements. For each measurement single stack images of at least twenty nuclei were taken, all with the same laser settings. The fluorescent intensity of the nuclei was determined with Image J (http://rsb.info.nih.gov/ij/). Each experiment was performed three times. CKS1 was used as neutral control.

### Microscopy

For fluorescence microscopy analyses, ovules were dissected from the pistil in 50 mM sodium phosphate buffer pH 7.5. YFP fluorescence of pollen and ovules at different developmental stages was analyzed on confocal microscopes (Leica TCS SP5 AOBS, Zeiss LSM 510 and Zeiss 710) using a BP 530–600 filter. For DIC microscopy, mature ovules and developing seeds were prepared from siliques before and after pollination, respectively, and mounted on microscope slides in a clearing solution of 8∶2∶1 chloral hydrate∶distilled water∶glycerol as described [9].

For DAPI staining, pollen grains were gently released into the DAPI solution (2.5 µg/ml DAPI, 0.01% Tween, 5% dimethyl sulfoxide, 50 mM Na phosphate buffer, pH 7.2) and incubated at 4°C overnight before observation. Pollen viability was assessed by mounting pollen as described [67]. DNA content was quantified and measured with the software ImageJ (http://rsbweb.nih.gov/ij) on images taken with constant settings as described [23]. Differential Interference Contrast (DIC) microscopy was done with an Axioimager (Zeiss).

### Chromatin immunoprecipitation

For ChIP experiments as described [12], [68], 2-week-old seedlings of plants expressing *Pro_RBR1_:RBR1:mRFP*, kindly provided by Dr. Frédéric Berger [38], growing on half-strength MS plates were used. Chromatin was sheared by means of a Bioruptor sonicator (Cosmo Bio) twice for 15 min with a 50% duty cycle and high-power output to obtain 200-bp to 1000-bp DNA fragments. A DsRed polyclonal antibody (Clontech) or an antibody raised against *Arabidopsis* E2FA protein, as kindly provided by Dr. Lieven De Veylder and described by Heyman et al. [69], together with Protein A-magnetic beads (Millipore) were used for immunoprecipitation. Negative controls were done without antibody. DNA was recovered with Magna ChIP spin filters according to the manufacturer's instructions (Millipore). ChIP DNA (0.5 µl or 1 µl of a 1/5 dilution) was analyzed by semi-quantitative PCR or quantitative real-time PCR with gene-specific primers, respectively (see Table S1). Three biological and three technical replicates were performed for ChIP quantitative PCR with PCNA1 as positive control and primers of the heterochromatic region as negative control.

### Expression analysis

Whole flowers (Figure 5) or dissected anthers (Figure 7) from the fifth and fourth floral buds preceding an open flower (containing mono- and bicellular pollen) were immediately frozen in liquid nitrogen and stored temporarily at −80°C. RNA was extracted using NucleoSpin RNA XS Kit (MACHEREY-NAGEL). RNA concentration and purity was tested using nanodrop-photometric quantification (Thermo Scientific). RNA integrity was verified by running 1 µl of total RNA on 1.5% agarose TBE-gels to detect the 28S and 16S rRNA bands. 100 ng up to 1 µg of total RNA was processed to obtain cDNA using polyT-primer and SuperScript III RNase H reverse transcriptase. As negative control, all steps were followed in the same manner, except for adding the reverse transcriptase. The resulting cDNA was used for quantitative Real Time-PCR (qRT-PCR) using the Roche LightCycler 480 system. Oligonucleotides were designed using either Primer3Plusdesign tool (http://www.bioinformatics.nl/cgi-bin/primer3plus/primer3plus.cgi) or QuantPrime (qPCR primer design tool: http://www.quantprime.de/main) and used in a final concentration of 0.5 µM each (primers are listed in Table S1). Three to four biological with three technical replicates each were processed. Cq calling was done using the Second Derivative Maximum method. Target specific efficiencies were calculated as the mean of all reaction specific efficiencies for a given target. Reaction specific efficiencies were deduced using LinRegPCR 7.4 (http://LinRegPCR.nl) [70], [71]. Data was quality controlled, normalized against 2 reference genes and statistically evaluated (unpaired t-test) using qbasePLUS 2.3 (http://www.biogazelle.com/products/qbaseplus) [72]. For the expression analysis of *FBL17* and *CDKA;1* in heterozygous *cdka;1* mutants, *ACT2* and *EF1 alpha* were used as reference genes. For the analysis of *FBL17* expression in *rbr1–2* mutants, *EF1 alpha* and *TIP41* were used as reference genes (see Table S1).

### Biomathematical simulations

The molecular network of G1/S regulators was described by ordinary differential equations. Phosphorylations/dephosphorylations and complex formations/dissociations were assumed to be fast, relative to protein synthesis and degradation, and steady-state approximations were used for these steps. The balance curves (nullclines) for total KRP and CDKA;1-cyclin complexes were calculated by the XPP/Aut program provided in Dataset S1.

## Supporting Information

Dataset S1 Source code of biomathematical simulation.
(DOC)

Figure S1 Depletion of CDKA;1 levels by amiCDKA;1. Western blot analysis of CDKA;1 abundance in the wild type and heterozygous *cdka;1^+/−^* mutants expressing a *PRO_CDKA;1_:amiCDKA;1* construct using an antibody directed against the PSTAIRE domain. Production of the amiRNA against *CDKA;1* can reduce protein levels in a wild-type background to approximately the level seen in heterozygous *cdka;1^+/−^* plants. Expression in a heterozygous *cdka;1^+/−^* mutant background can even further reduce protein levels. However, the *cdka;1* mutant pollen phenotype was only slightly enhanced in plants expressing the amiRNA construct (see Table 1).
(TIF)

Figure S2 *krp* mutant description. (A) The mutants in *KRP1*, *KRP2*, *KRP5*, *KRP6* and *KRP7* were previously described (see Material and Methods). (B) T-DNA insertion lines for *KRP3* and *KRP4* were newly obtained and, based on the absence of full-length transcripts, identified as null mutants.
(TIF)

Table S1 Primer sequences.
(XLS)

Text S1 Extended experimental procedures.
(DOC)
