# Supplementary material for: A General G1/S-Phase Cell-Cycle Control Module in the Flowering Plant Arabidopsis thaliana
Source: PLoS Genet. 2012 Aug 2;8(8):e1002847. doi: 10.1371/journal.pgen.1002847 (PMC3410867; doi:10.1371/journal.pgen.1002847)
Supplement: Dataset S1 — Source code of biomathematical simulation. (DOC) [file pgen.1002847.s001.doc]

**Supplementary data file S1** Source code of biomathematical simulation

# A simple XPP ode model for G1/S transition in plant cells

# DE for CdkA:cyclin complexes

CdkAT' = kscdka - kdcdka*CdkAT

# DE for the KRP CdkA inhibitors: constant synthesis and FBL17 mediated degradation

KRPT' = kskrp - (kdkrp' + kdkrp*FBL17)*KRPT

# steady state level of phosphorylated RbR

RbP = RbT*kprb*CdkA/(kprb*CdkA + kdprb)

# unphoshosphorylated RbR is total RbR minus phosphorylated

Rb = RbT - RbP

# steady state level of RbR:E2F complex assuming fast binding/unbinding

RBE2F = compl(Rb,E2FT,KdRE)

# free E2F is total minus the complex with RBbR

E2F = E2FT - RbE2F

# steady state level of FBL17: synthesis is E2F dependent

FBL17 = (ks17' + ks17*E2F)/kd17

# steady state level of the complex between CdkA:cyclin and KRP

CdkAKRP = compl(KRPT,CdkAT,kdAK)

# free CdkA:cyclin complex is total minus the complex with KRP

CdkA = CdkAT - CdkAKRP

# generic solution for a fast and reversible complex formation from two molecules

Trace(arg1,arg2,arg3)=arg1+arg2+arg3

compl(arg1,arg2,arg3)=2*arg1*arg2/(Trace(arg1,arg2,arg3)+sqrt(Trace(arg1,arg2,arg3)^2-4*arg1*arg2))

# parameters

p kskrp=0.01, kdkrp'=0.01, kdkrp=1

p kscdka=0.001, kdcdka=0.1

p RbT=2, kprb=1, kdprb=0.25, E2FT=1, KdRE=0.001

p ks17'=0, ks17=0.1,kd17=0.1, kdAK=0.01

done
