# Supplementary figures and images for: A General G1/S-Phase Cell-Cycle Control Module in the Flowering Plant Arabidopsis thaliana
Source: PLoS Genet. 2012 Aug 2;8(8):e1002847. doi: 10.1371/journal.pgen.1002847 (PMC3410867; doi:10.1371/journal.pgen.1002847)

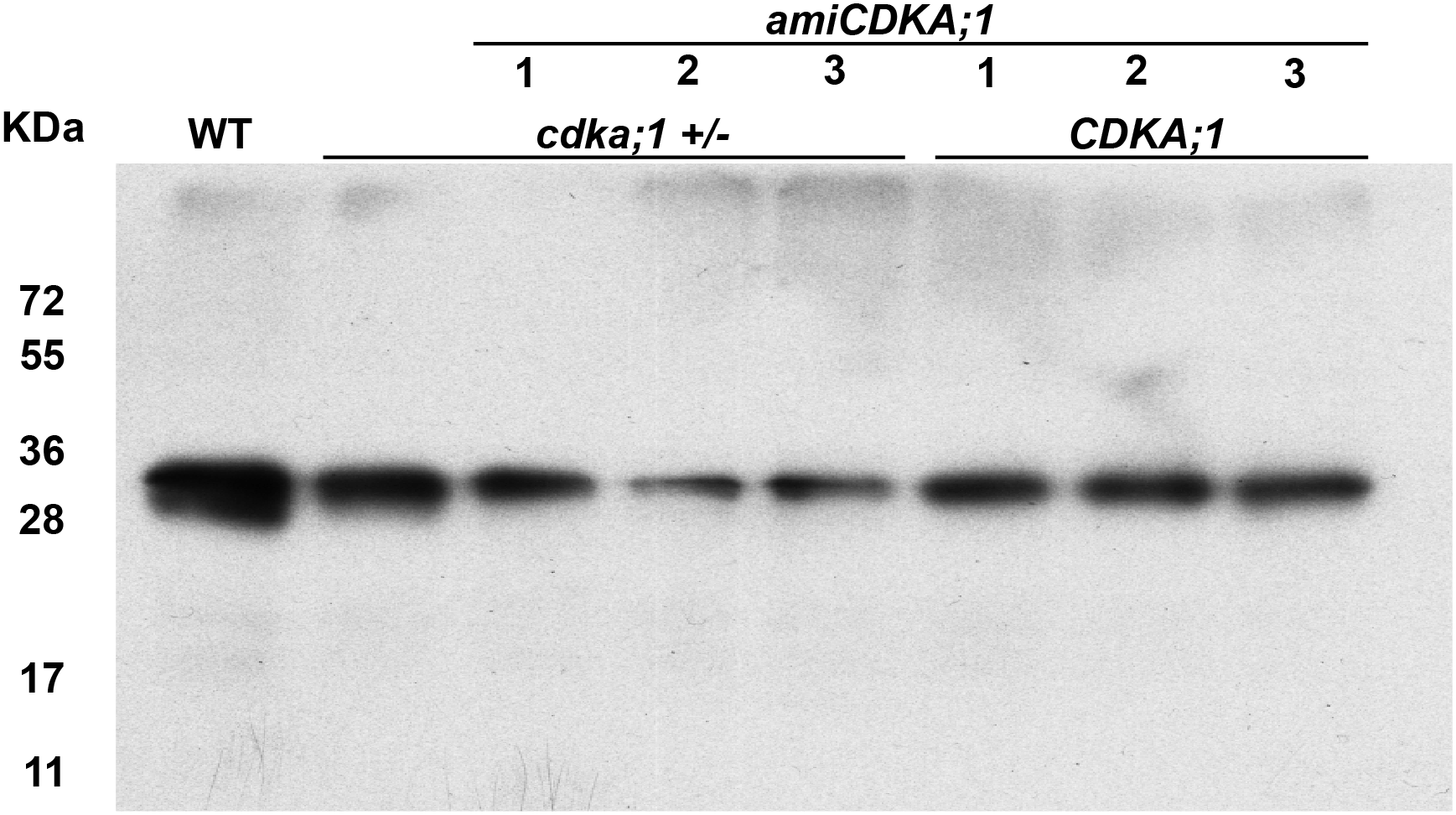

Supplement: Figure S1 — Depletion of CDKA;1 levels by amiCDKA;1. Western blot analysis of CDKA;1 abundance in the wild type and heterozygous cdka;1+/− mutants expressing a PROCDKA;1:amiCDKA;1 construct using an antibody directed against the PSTAIRE domain. Production of the amiRNA against CDKA;1 can reduce protein levels in a wild-type background to approximately the level seen in heterozygous cdka;1+/− plants. Expression in a heterozygous cdka;1+/− mutant background can even further reduce protein levels. However, the cdka;1 mutant pollen phenotype was only slightly enhanced in plants expressing the amiRNA construct (see Table 1). (TIF) [file pgen.1002847.s002.tif]

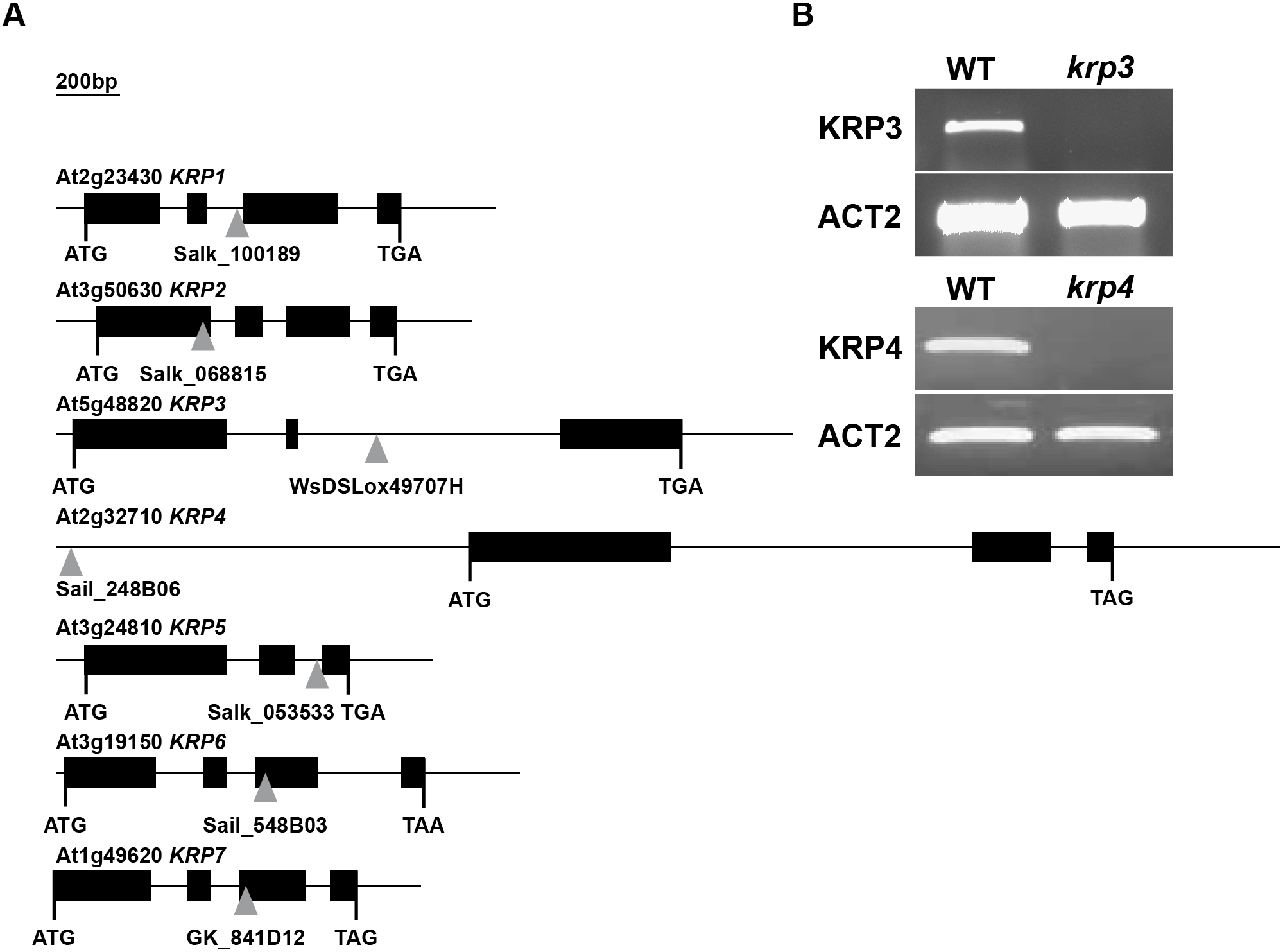

Supplement: Figure S2 — krp mutant description. (A) The mutants in KRP1, KRP2, KRP5, KRP6 and KRP7 were previously described (see Material and Methods). (B) T-DNA insertion lines for KRP3 and KRP4 were newly obtained and, based on the absence of full-length transcripts, identified as null mutants. (TIF) [file pgen.1002847.s003.tif]
