# Supplementary material for: A General G1/S-Phase Cell-Cycle Control Module in the Flowering Plant Arabidopsis thaliana
Source: PLoS Genet. 2012 Aug 2;8(8):e1002847. doi: 10.1371/journal.pgen.1002847 (PMC3410867; doi:10.1371/journal.pgen.1002847)
Supplement: Text S1 — Extended experimental procedures. (DOC) [file pgen.1002847.s005.doc]

**Extended experimental procedures**

Details on plant material and growth conditions

For mutants in *CDKA;1* (AT3G48750), the previously described allele *cdka;1-1* was used throughout [1]; for mutants in *FBL17* (At3g54650), the allele *fbl17-1* (GABI-170E02) [2]; for RBR1 (At3g12280), the allele *rbr1-2* (Salk_002946) [3]; and for *KRP6* (At3g19150), the allele *krp6-1* (Sail_548_B03) [2]. The CDKA;1-YFP plants had been described previously [4]. For the other mutants, the following lines were obtained from the stock center: *krp1* (At2g23430, Salk_100189), *krp2* (At3g50630, Salk_068815), *krp3* (At5g48820, WsDSLox49707H), *krp4* (At2g32710, Sail_248_B06), *krp5* (At3g24810, Salk_053533), *krp7* (At1g49620, GK_841D12), *e2fa* (At2g36010, GK_348E09).

Details on constructs and transformation

The CDKA;1 variants *CDKA;1^D146N^* and *CDKA;1^PSTAIRE-dead^* were constructed by fusion PCR on *Arabidopsis CDKA;1* wild-type cDNA and flanked by the Gateway sites attB1 and attB2 for recombination in pDONR201 (Invitrogen). For *CDKA;1^D146N^* and *CDKA;1^PSTAIRE-dead^*, the primer combinations used were ND10/ND161 and ND11/ND160 and ND10/ND251 and ND11/ND250, respectively. Fragments were fused in a final PCR with ND10/ND11. Primer sequences are listed in Table S1. The obtained Gateway entry clones were recombined into the destination vector pAM-PAT-GW-ProCDKA;1 [1]. To generate *Strep-tag III* *fused CDKA;1^K33R^*, a site-directed mutagenesis was conducted with a *StrepIII-CDKA;1* cDNA cloned into pDONR223 (Invitrogen) [5] as a template of the inverse PCR with primers #361 and #362. The PCR product was phosphorylated with T4 polynucleotide kinase (Fermentas) and ligated with T4 DNA ligase (Fermentas). After confirmation of the sequence, a recombination reaction was done between the Gateway entry clone and the destination vector pAM-PAT-GW-ProCDKA;1 by means of the LR Clonase II (Invitrogen). A Gateway entry clone with the FBL17 cDNA [2] was recombined into the destination vector pAM-PAT-GW-ProUBQ and pAM-PAT-GW-ProCDKA;1. The pAM-PAT-GW-ProUBQ vector was constructed by inserting the Ubiquitin promoter (kindly provided by Niko Geldner, [6]) into the pAM-PAT-GW vector by restriction-ligation procedure. Artificial micro-RNAs (amiRNAs) against CDKA;1 were designed and cloned as described [7] with the plasmid pRS300 (kindly provided by Detlef Weigel) as a PCR template (http://wmd.weigelworld.org/cgi-bin/mirnatools.pl). Primers A and B contained the attB1 and attB2 Gateway recombination site, respectively. amiRNA sequence and primers used are indicated in Supplemental Table S1. The PCR product was cloned into the Gateway entry vector pDONR201 (Invitrogen) with the BP Clonase II (Invitrogen). After confirmation of the sequence, a recombination reaction was done between the entry clone and the destination vector pAM-PAT-GW-ProCDKA;1 with the LR Clonase II (Invitrogen). All resulting binary plant expression vectors, that conferred phosphinothricin (BASTA; Bayer Cropscience) resistance, were retransformed into *Agrobacterium tumefaciens* strain GV3101-pMP90RK [8] and heterozygous plants for *cdka;1*^+/-^ were transformed.

References

1. Nowack MK, Grini PE, Jakoby MJ, Lafos M, Koncz C, Schnittger A (2006) A positive signal from the fertilization of the egg cell sets off endosperm proliferation in angiosperm embryogenesis. Nat Genet 38: 63-67.

2. Gusti A, Baumberger N, Nowack M, Pusch S, Eisler H, Potuschak T, De Veylder L, Schnittger A, Genschik P (2009) The Arabidopsis thaliana F-box protein FBL17 is essential for progression through the second mitosis during pollen development. PLoS ONE 4: e4780.

3. Ingouff M, Jullien P, Berger F (2006) The female gametophyte and the endosperm control cell proliferation and differentiation of the seed coat in Arabidopsis. The Plant Cell 18: 3491-3501.

4. Nowack MK, Shirzadi R, Dissmeyer N, Dolf A, Endl E, Grini PE, Schnittger A (2007) Bypassing genomic imprinting allows seed development. Nature 447: 312-315.

5. Pusch S, Harashima H, Schnittger A (accepted) Identification of kinase substrates by bimolecular complementation assays. Plant Journal

6. Geldner N, Denervaud-Tendon V, Hyman DL, Mayer U, Stierhof YD, Chory J (2009) Rapid, combinatorial analysis of membrane compartments in intact plants with a multicolor marker set. Plant J 59: 169-178.

7. Schwab R, Ossowski S, Riester M, Warthmann N, Weigel D (2006) Highly Specific Gene Silencing by Artificial MicroRNAs in Arabidopsis. Plant Cell

8. Koncz C, Schell J (1986) The promotor of T_L-DNA_ gene 5 controls the tissue-specific expression of chimaeric genes carried by a noval Agrobacterium binary vector. Mol Gen Genet 204: 383-396.
